# Biochar amendment alters root morphology of maize plant: Its implications in enhancing nutrient uptake and shoot growth under reduced irrigation regimes

**DOI:** 10.3389/fpls.2023.1122742

**Published:** 2023-01-20

**Authors:** Heng Wan, Xuezhi Liu, Qimiao Shi, Yiting Chen, Miao Jiang, Jiarui Zhang, Bingjing Cui, Jingxiang Hou, Zhenhua Wei, Mohammad Anwar Hossain, Fulai Liu

**Affiliations:** ^1^ Key Laboratory of Agricultural Soil and Water Engineering in Arid and Semiarid Areas, Ministry of Education, Northwest A&F University, Yangling, Shaanxi, China; ^2^ College of Water Resources and Architectural Engineering, Northwest A&F University, Yangling, Shaanxi, China; ^3^ School of Civil and Hydraulic Engineering, Ningxia University, Yinchuan, China; ^4^ Department of Plant and Environmental Sciences, Faculty of Science, University of Copenhagen, Taastrup, Denmark; ^5^ Department of Genetics and Plant Breeding, Bangladesh Agricultural University, Mymensingh, Bangladesh; ^6^ Sino-Danish Center for Education and Research, University of Chinese Academy of Sciences, Beijing, China

**Keywords:** biochar, alternate partial root-zone drying irrigation, soil available nutrient, root morphology, biomass, nutrient uptake

## Abstract

**Introduction:**

Biochar amendment provides multiple benefits in enhancing crop productivity and soil nutrient availability. However, whether biochar addition affects root morphology and alters plant nutrient uptake and shoot growth under different irrigation regimes remain largely unknown.

**Methods:**

A split-root pot experiment with maize (*Zea mays* L.) was conducted on clay loam soil mixed with 2% (w/w) of wheat-straw (WSP) and softwood (SWP) biochar. The plants were subjected to full (FI), deficit (DI), and alternate partial root-zone drying (PRD) irrigation from the fourth leaf to the grain-filling stage.

**Results and discussion:**

The results showed that, compared to plants grown in unamended soils, plants grown in the biochar-amended soils possessed greater total root length, area, diameter, volume, tips, forks, crossings, and root length density, which were further amplified by PRD. Despite a negative effect on soil available phosphorus (P) pool, WSP addition improved soil available nitrogen (N), potassium (K), and calcium (Ca) pool and cation exchange capacity under reduced irrigation. Even though biochar negatively affected nutrient concentrations in shoots as exemplified by lowered N, P, K (except leaf), and Ca concentration, it dramatically enhanced plant total N, P, K, Ca uptake, and biomass. Principal component analysis (PCA) revealed that the modified root morphology and increased soil available nutrient pools, and consequently, the higher plant total nutrient uptake might have facilitated the enhanced shoot growth and yield of maize plants in biochar-added soils. Biochar amendment further lowered specific leaf area but increased leaf N concentration per area-to-root N concentration per length ratio. All these effects were evident upon WSP amendment. Moreover, PRD outperformed DI in increasing root area-to-leaf area ratio. Overall, these findings suggest that WSP combined with PRD could be a promising strategy to improve the growth and nutrient uptake of maize plants.

## Graphical Abstract

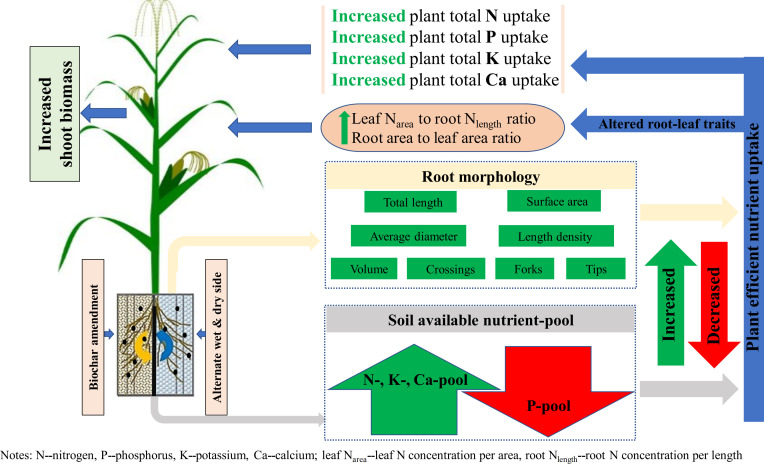

## 1 Introduction

Global climate change, soil degradation, and shortage of freshwater resources adversely affect agricultural productivity (Chen et al., 2013). Low soil fertility and water scarcity constrain crop yield (Reich et al., 2003). In order to improve agricultural productivity and sustainability, novel management strategies aiming at improving soil fertility and crop water-use efficiency need to be developed.

As an effective soil amendment, biochar has received widespread concerns in terms of increasing soil water holding capacity, soil carbon (C) sequestration, and nutrient bioavailability (Atkinson et al., 2010). Generally, biochar properties including pH, EC (electrical conductivity), CEC (cation exchange capacity), total ash, and nutrient content differ wildly between different feedstocks and could create multiple impacts on the rhizosphere environment (Liu et al., 2022). For instance, application of crop residue-derived biochar could increase soil total nitrogen (N) and available phosphorus (P) and potassium (K) content, contributing to nutrient uptake and growth of plants (Oladele et al., 2019). Such results concur with our previous study reporting that wheat-straw biochar yielded significant positive effects on soil nutrient availability, resulting in better K uptake and growth of tobacco (Liu et al., 2021). In contrast to these, some previous studies have noted that lignocellulose-containing wood-based biochar amendment inhibited N and ortho−P bioavailability in soil due to its anti-decomposition capacity and initial low CEC (Gul et al., 2015; Chen et al., 2021). Moreover, alteration of soil nutrient availability could be caused by adsorption/desorption processes occurring on biochar surface and precipitation/dissolution of minerals derived from biochar, thereby modulating soil nutrient pool and nutrient bioavailability (Bornø et al., 2019).

Partial root-zone drying (PRD) irrigation, an improvement on conventional deficit irrigation, employs a spatiotemporal strategy of soil wetting/drying cycles, which could positively stimulate soil microbial activity and respiration rate, thereby influencing the mineralization fixation as well as the turnover process of soil nutrients (Xiang et al., 2008; Wang et al., 2010). Furthermore, the re-wetting process in dry soil could alter soil structure properties (Rahman et al., 2018), like water absorption ability and swelling of soil aggregates, which may facilitate the decomposition of organic matter and accelerate the mineralization of mineral elements (Miller et al., 2005), hereby improving soil nutrient bioavailability (Liu et al., 2021). Such effect is known as the “Birch effect” (Birch, 1958).

Both biochar amendment and altered water dynamics under the PRD irrigation could alter the growth and physiology of plant. For instance, Liu et al. (2022) demonstrated that biochar amendment could effectively enhance root biomass density but decreased root average diameter; Xiang et al. (2017) showed that root biomass, root length, and root tip number could be increased upon the application of biochar, strengthening the ability of plants to access resources. Similarly, PRD irrigation could increase the number of secondary root and root length (Liu et al., 2020; Qi et al., 2020). It has been demonstrated that repeated drying of the root zone could stimulate thin root growth and maximize nutrient and water availability (Comas et al., 2013). Yet, previous studies have paid less attention to the combined effects of biochar and PRD on root morphology, nutrient uptake, and their implications in altering shoot growth. Nonetheless, earlier studies have showed that biochar amendment and PRD irrigation could modulate shoot growth and leaf morphology. For instance, specific leaf area (SLA) was lowered in plants under both biochar amendment and drought conditions (Olmo et al., 2014; Liu et al., 2022). The decline in SLA was probably caused by differences in the sensitivity of photosynthesis and leaf area expansion to the change of growth environment, with leaf expansion likely to be more susceptible (Liu and Stützel, 2004; Jensen et al., 2010). It has been suggested that change of root morphology could directly influence the shoot growth as there are obvious allometric relationships between the aboveground and belowground traits that are responsive to the growth conditions of plant (Freschet et al., 2010; Silva et al., 2018). Li and Bao (2015) found that shoot height and biomass differences are closely related to the total root length. The specific root length has been described to vary considerably among soil types and negatively correlated to SLA (Liu et al., 2022). Such relationship between root morphology and leaf development may have a direct consequence on plant photosynthetic capacity, nutrient demands, and adaptability to extreme environments (Dong et al., 2020). However, to date, how PRD irrigation and biochar amendment affect root growth and its consequence on altering plant nutrient uptake and shoot development remain unclear.

The aim of the present study was to explore the combined effects of different biochar (mixed softwood and wheat-straw biochar) amendments and PRD irrigation on the root morphology, plant nutrient uptake, and shoot growth of maize plants. It was hypothesized that biochar amendment would (1) enhance soil available nutrient content, (2) alter plant morphological traits and their allometric relationships, and (3) increase plant biomass and nutrient uptake under the PRD regime.

## 2 Materials and methods

### 2.1 Soil and biochar

Two biochar materials, wheat-straw (WSP) and mixed softwood (SWP) pellet biochar, were obtained from the UK Biochar Research Centre (UKBRC), UK. Both biochars were produced *via* pyrolysis at 550°C. The pelletized biochars were crushed and passed over a 0.45-mm screen. The clay loam soil was obtained from the field in Yangling and sieved through a 5-mm sieve after air-drying. The soil water contents at full pot water-holding capacity and the permanent wilting point were 30% and 5%, respectively, and the bulk density was 1.30 g cm^−3^. The soil and biochar properties are presented in **Table 1**.

**Table 1:** Soil and biochar properties.

Factor	Soil	SWP	WSP
Clay (<0.002 mm, %)	8	–	–
Silt (0.002–0.05 mm, %)	85	–	–
Sand (0.05–2 mm, %)	7	–	–
pH	7.7	7.9	9.9
EC (μS cm^−1^)	360	90	1700
CEC (cmol + kg^−1^)	2.0	3.2	6.2
Total C (%)	1.8	85.5	68.3
Total N (%)	0.1	<0.1	1.4
Total P^(c)^ (%)	0.1	0.1	0.1
Total K^(c)^ (%)	2.4	0.3	1.6
Total Ca^(c)^ (%)	7.4	0.3	0.8
C:N	18	<855.2	49.1
H:C_tot_	–	0.4	0.4
O:C_tot_	–	0.1	0.1
(O + N):C		< 0.1	0.1
Surface area (m^2^ g^−1^)	–	26.4	26.4
Total ash^(a)^ (%)	–	1.3	21.2
C stability^(b)^ (%)	–	69.6	96.5

^(a)^TGA; ^(b)^Cross A, Sohi SP (2013); ^(c)^Aqua Regia digestion followed by ICP.

### 2.2 Experimental setup

The pot experiment with maize (*Zea mays* L.) plants was carried out in a greenhouse located at the Northwest A&F University, Yangling, Shaanxi province, China (34° 15′N, 108° 04′E). The plants were grown in split-root pots (8 L) filled with 9.0 kg of clay loam soil mixed with 2% (w/w) of either WSP or SWP biochar, and pots with soil without biochar addition were set as controls. To ensure the nutrient requirement for plant growth during the experiment, 2 g N, 2 g P, and 0.22 g K were applied into all pots. The maize seeds were sown in peat on 4 March 2021 with the seedlings being transplanted into the pots at the four-leaf stage. After transplanting, the pots were daily irrigated to 90% of water-holding capacity for 30 days; thereafter, three irrigation regimes were implemented: FI (full irrigation), the whole pots were daily irrigated to 90% of water-holding capacity; DI (deficit irrigation), the whole pots were daily irrigated with 70% volume of water used in FI; and PRD (partial root-zone drying irrigation), the amount of irrigation on one compartment is the same as the DI, and the irrigation was switched while the soil water content of the other compartment decreased to 10%–12%.

The soil water content was measured by a time domain reflectometer (TDR, TRASE, Soil Moisture Equipment Crop., Goleta, CA, USA) at 4:00 p.m. each day to supplement the water consumption of the previous day. At the onset of the irrigation treatments, a 2-cm layer of perlite was placed on the soil surface to minimize soil evaporation. The experiment lasted for 9 weeks, during which each soil compartment of the PRD-treated pots was subjected to three drying/wetting cycles. Maize plants were harvested twice: one harvest before starting the irrigation treatments and the final harvest at the end of the irrigation treatment on day 63.

### 2.3 Leaf area, dry biomass, and nutrient accumulation in plant organs

At both harvests, leaf area (LA) was measured with a LI-3100 portable leaf area meter (LI-Cor, Inc. Lincoln, NE, USA). Specific leaf area (SLA, cm^2^ g^−1^) was calculated using LA divided by leaf dry matter (LDM). The leaves, stems, fruits, and roots were harvested separately. The plant samples were dried in an oven at 105°C for 30 min, then at 75°C 48 h to constant weight, and recorded as LDM, stem dry matter (S_tem_DM), fruit dry matter (FDM), shoot dry matter (S_hoot_DM), and root dry matter (RDM). The root-to-shoot dry biomass ratio was also calculated. Dry plant samples were ground into fine powder and used to analyze nutrient concentration. Nitrogen concentration ([N]) in leaves, stems, and roots was determined using an Elemental Analyzer (Vario Isotope Cube, Hanau Elementar, Germany). Leaf N_area_ ([N] per leaf area)-to-root N_length_ ([N] per root length) ratio was calculated according to the method described by Liu et al. (2010). The concentrations of phosphorus ([P]), potassium ([K]), and calcium ([Ca]) in leaves, stems, and roots were determined by inductively coupled plasma mass spectrometry (ICP-MS, Agilent 7700×, Agilent Technologies, USA). Plant total nutrient accumulation, i.e., [PTN], [PTP], [PTK], and [PTCa], was calculated as:


$$PTN={\left[N\right]}_{leaf}\times leaf\;dry\;matter+{\left[N\right]}_{stem}\times stem\;dry\;matter+{\left[N\right]}_{root}\times root\;dry\;matter$$

$$PTP={\left[P\right]}_{leaf}\times leaf\;dry\;matter+{\left[P\right]}_{stem}\times stem\;dry\;matter+{\left[P\right]}_{root}\times root\;dry\;matter$$

$$PTK={\left[K\right]}_{leaf}\times leaf\;dry\;matter+{\left[K\right]}_{stem}\times stem\;dry\;matter+{\left[K\right]}_{root}\times root\;dry\;matter$$

$$PTCa={\left[Ca\right]}_{leaf}\times leaf\;dry\;matter+{\left[Ca\right]}_{stem}\times stem\;dry\;matter+{\left[Ca\right]}_{root}\times root\;dry\;matter$$

### 2.4 Root morphological traits

Root traits were analyzed and calculated with reference to Liu et al. (2022) and Li et al. (2009). Briefly, the cleaned root samples were placed on 20 × 25 cm transparent trays with a bottom layer covered with deionized water to avoid stacking the roots together. Subsequently, root samples were scanned at 400 dots per inch by a photo flatbed scanner (EPSON Perfection V700, Epson America, Inc.). The resulting images were analyzed by WinRHIZO Pro root analysis software (Version 2012b; Regent Instruments Inc., Québec City, QC, Canada) for root morphological traits, including total root length (RL), root surface area (RA), root average diameter (RD), root volume (RV), root tips (RT), root forks (RF), and root crossings (RC). Specific root length (SRL), specific root area (SRA), and specific root volume (SRV) were calculated by dividing RL by RDM, RA by RDM, and RV by RDM, respectively. Root length density (RLD; RL per unit soil volume), root tissue density (RTD; RL per unit RV), and RA-to-LA ratio (RLR) were also calculated.

### 2.5 Determination of soil nutrient contents

Soil samples collected from the rhizosphere of maize plants were air-dried, sieved through a 1-mm screen. To determine soil available nitrogen (SAN) content, 2 g of soil sample was extracted in 10.0 ml of 1.8 M NaOH solution, and the SAN was determined by the digital burette method (Brand titrette, Germany) where 2 ml of 2% H_3_BO_3_ was used as an indicator. For analyzing soil available phosphorus (SAP) content, 2.5 g of soil sample was extracted in 50.0 ml of 0.5 M NaHCO_3_ solution (pH 8.5), and the SAP was determined by a UV-Visible spectrophotometer (UV-2450, Shimadzu, Japan) at 880 nm. To measure soil available potassium (SAK) content, 5 g of soil sample was digested with 50 ml of 1 M NH_4_OAc solution (pH 7.0), and the SAK was determined by a flame photometer (PFP7; Jenway, UK). For soil exchange calcium (SECa) determination, 5 g of soil sample was extracted in 50 ml of NH_4_Cl–70% C_2_H_5_OH solution (pH 8.5), and the SECa was determined by an atomic absorption spectrophotometer (Z-2000, Hitachi, Japan). Soil cation exchange capacity (CEC) was determined by the HCl-Ca(OAc)_2_ extraction method at pH 8.2. The size of soil available nutrient pools was calculated as the sum of soil available or exchange nutrients and plant total nutrient accumulation.

### 2.6 Statistical analysis

Two-way analysis of variance (ANOVA) was carried out showing the effect of the biochar ([B]) and irrigation regime ([I]), as well as their interaction, i.e., [B] × [I]. Further one-way ANOVA and Tukey’s multiple range tests with a 5% confidence level were applied when there was a significant interaction between the independent variables. ANOVA was conducted with IBM SPSS Statistics 23 (SPSS Inc., New York, USA) and the significance analysis of correlation was assessed using Pearson’s product-moment correlation. Pearson correlation was used to evaluate the relationships between aboveground and belowground variables *via* the correlation heatmap of genescloud tool (https://www.genescloud.cn). Principal component analysis (PCA) was further performed on all the parameters by the ORIGIN-Pro 2021 software (OriginLab Inc., Northampton, Massachusetts, USA).

## 3 Results

### 3.1 Root morphological traits

**Table 2** shows that total RL, RA, RD, RV, RT, RF, and RC of maize plants were significantly greater under biochar compared to non-biochar controls. RLD was solely affected by biochar treatment (**Table 3**), being greater for biochar-amended plants than for non-biochar plants. All these effects of biochar on root morphological traits were more evident with WSP. However, SRL, RTD, SRA, and SRV were not affected (**Table 3**). Interestingly, there was a clear tendency that reduced irrigation treatments increased RTD in relation to FI (**Table 3**).

**Table 2:** The effects of treatments and output of two-way ANOVA for total root length (RL), area (RA), diameter (RD), volume (RV), tips (RT), forks (RF), and crossings (RC) of maize plants.

Biochar (B)	Irrigation (I)	RL (m)	RA (cm^2^)	RD (mm)	RV (cm^3^)	RT (k plant^-1^)	RF (k plant^-1^)	RC (k plant^-1^)
Control	FI	48.53 ± 13.46	627.03 ± 197.56	2.37b ± 0.43	8.81d ± 2.30	14.29 ± 4.96	23.78 ± 10.88	3.18 ± 16.11
	PRD	66.06 ± 11.19	912.34 ± 105.35	3.22b ± 0.40	10.59cd ± 0.79	25.61 ± 5.12	35.36 ± 4.93	5.31 ± 10.59
	DI	73.81 ± 4.60	1,007.20 ± 72.38	3.36ab ± 0.49	11.43cd ± 0.88	26.72 ± 0.80	41.20 ± 2.76	6.54 ± 0.75
SWP	FI	71.66 ± 9.87	1,290.32 ± 131.13	4.50ab ± 0.45	19.43abc ± 1.60	27.28 ± 5.04	43.79 ± 7.93	5.28 ± 1.13
	PRD	90.24 ± 15.07	1,327.84 ± 150.09	4.05ab ± 0.46	17.21bcd ± 1.68	27.00 ± 4.80	52.12 ± 9.88	8.48 ± 2.25
	DI	57.53 ± 14.59	873.16 ± 181.53	2.47b ± 0.48	11.03cd ± 1.90	22.25 ± 1.43	35.23 ± 5.94	5.57 ± 1.20
WSP	FI	116.25 ± 22.15	1,989.28 ± 309.38	5.90a ± 1.04	28.79a ± 3.62	39.95 ± 5.68	67.86 ± 12.21	8.63 ± 2.12
	PRD	134.87 ± 17.64	1,939.19 ± 233.92	4.64ab ± 0.64	24.12ab ± 2.91	41.83 ± 5.02	81.79 ± 10.03	14.31 ± 0.02
	DI	99.10 ± 25.95	1,458.74 ± 260.55	3.58ab ± 0.32	18.71abcd ± 2.05	32.54 ± 8.16	60.04 ± 15.92	9.87 ± 3.37
Output of two-way ANOVA (*p*-value)								
Biochar (B)		***	***	**	***	**	***	**
Irrigation (I)		ns	ns	ns	ns	ns	ns	ns
B * I		ns	ns	*	*	ns	ns	ns

The treatments are different biochar (Control, SWP, and WSP) and irrigation (FI, DI, and PRD).

Values are the mean ± standard error (n = 4). *, **, and *** indicate significant levels at p< 0.05, p< 0.01, and p< 0.001, respectively. ns indicates no statistical significance. Different letters following the mean indicate significant differences between treatments at the p< 0.05 level by Tukey’s test. k stands for 1000.

**Table 3:** The effects of treatments and output of two-way ANOVA for specific root length (SRL), root length density (RLD), root tissue density (RTD), specific root area (SRA), and specific root volume (SRV) of maize plants.

Biochar (B)	Irrigation (I)	SRL (m g^−1^)	RLD (m L^−1^)	RTD (g cm^−3^)	SRA (cm^2^ g^−1^)	SRV (cm^3^ g^−1^)
Control	FI	17.82 ± 1.64	4.71 ± 1.73	0.32 ± 0.07	247.49 ± 48.28	3.50 ± 0.54
	PRD	19.70 ± 1.79	8.26 ± 1.40	0.33 ± 0.06	283.25 ± 38.16	3.43 ± 0.68
	DI	18.60 ± 2.96	9.23 ± 0.58	0.38 ± 0.07	254.48 ± 42.66	2.89 ± 0.49
SWP	FI	12.28 ± 0.33	8.96 ± 1.23	0.30 ± 0.03	224.80 ± 9.03	3.44 ± 0.29
	PRD	16.27 ± 2.13	11.28 ± 1.88	0.32 ± 0.03	242.23 ± 12.78	3.19 ± 0.30
	DI	15.42 ± 3.18	7.19 ± 1.82	0.36 ± 0.03	239.72 ± 46.28	3.08 ± 0.59
WSP	FI	14.31 ± 1.68	14.53 ± 2.77	0.28 ± 0.03	248.41 ± 21.46	3.65 ± 0.29
	PRD	17.90 ± 0.98	16.86 ± 2.20	0.31 ± 0.02	258.69 ± 16.39	3.22 ± 0.23
	DI	16.31 ± 2.91	12.39 ± 3.24	0.32 ± 0.03	247.76 ± 27.11	3.27 ± 0.36
Output of two-way ANOVA (*p*-value)						
Biochar (B)		ns	***	ns	ns	ns
Irrigation (I)		ns	ns	ns	ns	ns
B * I		ns	ns	ns	ns	ns

The treatments are different biochar (Control, SWP, and WSP) and irrigation (FI, DI, and PRD).

Values are the mean ± standard error (n = 4). *** indicates significant levels at p< 0.001. ns indicates no statistical significance.

### 3.2 Soil available nutrient pools and cation exchange capacity status

**Figure 1:**
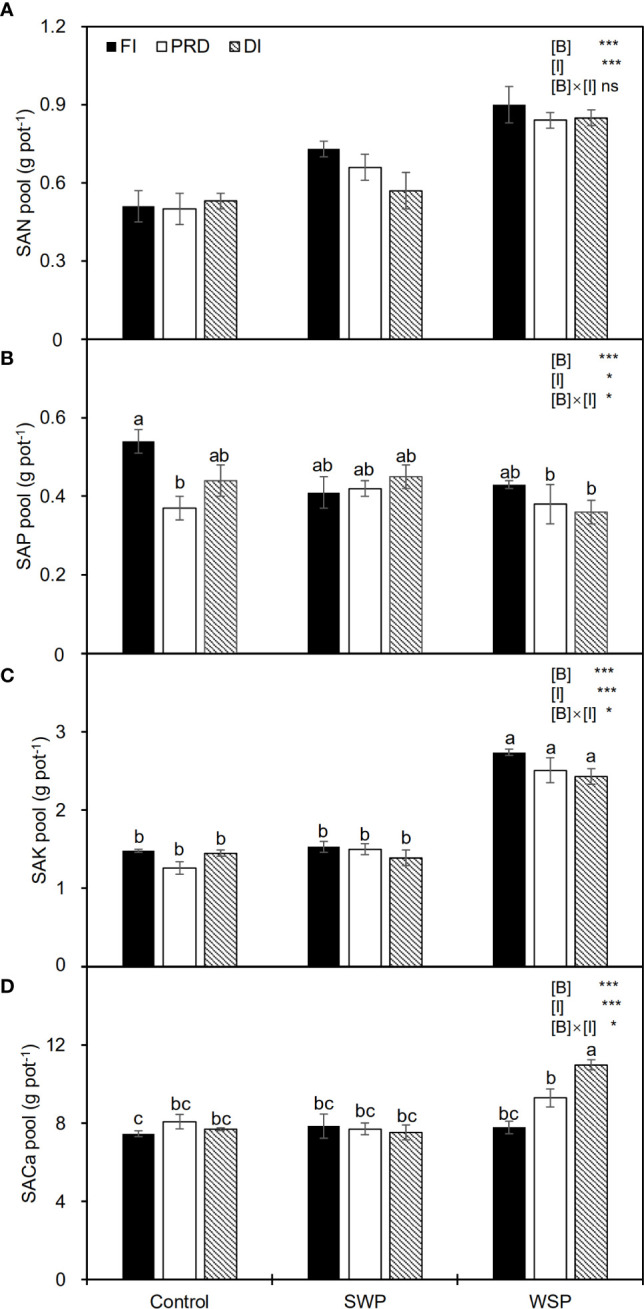
**(A)** Soil available N pool (SAN pool), **(B)** soil available P pool (SAP pool), **(C)** soil available K pool (SAK pool), and **(D)** soil available Ca pool (SACa pool) of maize plants exposed to different biochar (Control, SWP and WSP) and irrigation (FI, DI, and PRD) treatments. Values are the mean ± standard error (*n* = 4). * and *** indicate significant levels at *p*< 0.05 and *p*< 0.001, respectively. ns indicates no statistical significance. Different letters in the bars indicate significant differences between treatments at the *p*< 0.05 level by Tukey’s test.

**Table 4** showed the effects of biochar and irrigation treatment on soil available nutrients. SAN was not affected by either biochar addition or irrigation treatment. It was found that SAP was solely affected by biochar addition, where a significant reduction in SAP was found in the biochar treatments, particularly with the addition of WSP. However, there was an interaction between biochar addition and irrigation treatment on SAP. The trend for SAK and SECa to be affected by biochar addition was consistent, both having the highest values in the WSP. However, in the irrigation treatment, compared to FI, reduced irrigation significantly decreased SAP but increased SAK. In addition, there was a significant two-way interaction on SECa. SAN pool was significantly affected by two principal factors (**Figure 1A**), viz., biochar amendment, particularly WSP, increased SAN pool compared to unamended soils; regardless of biochar amendment, FI treatment had greater SAN pool than did reduced irrigation, and the SAN pool under PRD was slightly higher concerning DI (**Figure 1A**). However, compared to the unamended soils, biochar amendment, particularly WSP, remarkably decreased SAP pool (**Figure 1B**). Reduced irrigation especially PRD reduced SAP pool compared to FI (**Figure 1B**). SAK pool was significantly increased by WSP addition compared to the unamended soils (**Figure 1C**). Regardless of biochar amendment, FI led to greater SAK pool in relation to the reduced irrigation treatments (**Figure 1C**). Likewise, WSP significantly increased SACa pool than the unamended soils (**Figure 1D**). Compared to FI, reduced irrigation significantly increased SACa pool (**Figure 1D**). Moreover, there were interactive effects between biochar addition and irrigation treatment on SAP, SAK, and SACa pool (**Figures 1B–D**)

**Table 4:** The effects of treatments and output of two-way ANOVA for soil available N (SAN), soil available P (SAP), soil available K (SAK), and soil exchange Ca (SECa) content.

Biochar (B)	Irrigation (I)	SAN (g pot^−1^)	SAP (g pot^−1^)	SAK (g pot^−1^)	SECa (g pot^−1^)
Control	FI	0.14 ± 0.01	0.47a ± 0.02	1.22 ± 0.04	7.34c ± 0.16
	PRD	0.13 ± 0.01	0.30b ± 0.04	0.97 ± 0.09	7.96bc ± 0.36
	DI	0.14 ± 0.01	0.37ab ± 0.04	1.13 ± 0.05	7.57bc ± 0.08
SWP	FI	0.13 ± 0.00	0.29b ± 0.03	1.06 ± 0.0 6	7.67bc ± 0.63
	PRD	0.14 ± 0.01	0.33ab ± 0.01	1.09 ± 0.06	7.56bc ± 0.30
	DI	0.13 ± 0.00	0.36ab ± 0.03	1.00 ± 0.07	7.41bc ± 0.37
WSP	FI	0.12 ± 0.01	0.28b ± 0.02	2.10 ± 0.08	7.55bc ± 0.32
	PRD	0.13 ± 0.01	0.25b ± 0.05	1.88 ± 0.14	9.10ab ± 0.47
	DI	0.14 ± 0.01	0.23b ± 0.03	1.74 ± 0.08	10.80a ± 0.26
Output of two-way ANOVA (*p*-value)					
Biochar (B)		ns	***	***	***
Irrigation (I)		ns	ns	*	**
B * I		ns	*	ns	***

The treatments are different biochar (Control, SWP, and WSP) and irrigation (FI, DI, and PRD).

Values are the mean ± standard error (n = 4). *, **, and *** indicate significant levels at p< 0.05, p< 0.01, and p< 0.001, respectively. ns indicates no statistical significance. Different letters following the mean indicate significant differences between treatments at the p< 0.05 level by Tukey’s test.

**Figure 2:**
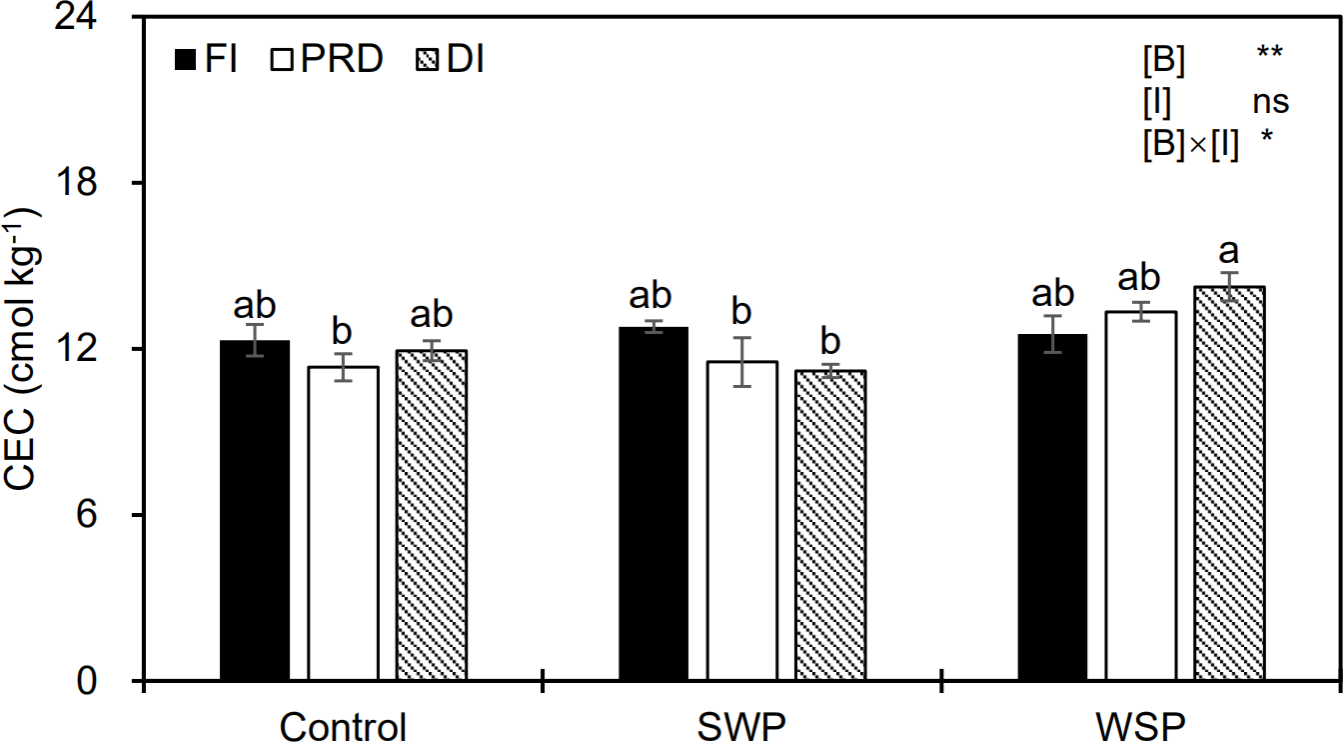
Soil cation exchange capacity (CEC) status of maize plants exposed to different biochar (Control, SWP, and WSP) and irrigation (FI, DI, and PRD) treatments. Values are the mean ± standard error (*n* = 4). * and ** indicate significant levels at *p*< 0.05 and *p*< 0.01, respectively. ns indicates no statistical significance. Different letters in the bars indicate significant differences between treatments at the *p*< 0.05 level by Tukey’s test.

Soil CEC varied among biochar treatments (**Figure 2**), being greater for biochar amendment than for unamended soils. Although irrigation treatment alone had no obvious effect on CEC, a significant interactive effect between the main factors on CEC was observed (**Figure 2**).

### 3.3 Nutrient acquisition in plant organs and whole plants

Nutrient concentrations for each organ of maize plants are shown in **Table 5**. Both [N]_leaf_ and [N]_root_ were significantly influenced by biochar addition and irrigation treatment; [N]_stem_ was significantly affected only by biochar amendment. Interestingly, regardless of irrigation treatment, biochar amendment decreased N concentration in all organs relative to the unamended controls. Across the biochar treatments, reduced irrigation increased [N]_leaf_ and [N]_root_ than did FI, and PRD had greater value than that of DI treatment while the [N]_root_ was the opposite.

**Table 5:** The effects of treatments and output of two-way ANOVA for [N]_leaf_, [N]_stem_, [N]_root_, [P]_leaf_, [P]_stem_, [P]_root_, [K]_leaf_, [K]_stem_, [K]_root_, [Ca]_leaf_, [Ca]_stem_, and [Ca]_root_ of maize organs.

Biochar (B)	Irrigation (I)	[N]_leaf_ (g kg^−1^)	[N]_stem_ (g kg^−1^)	[N]_root_ (g kg^−1^)	[P]_leaf_ (g kg^−1^)	[P]_stem_ (g kg^−1^)	[P]_root_ (g kg^−1^)	[K]_leaf_ (g kg^−1^)	[K]_stem_ (g kg^−1^)	[K]_root_ (g kg^−1^)	[Ca]_leaf_ (g kg^−1^)	[Ca]_stem_ (g kg^−1^)	[Ca]_root_ (g kg^−1^)
Control	FI	25.64 ± 0.35	18.96 ± 1.43	18.88 ± 1.44	5.34 ± 0.50	4.07a ± 0.16	2.35 ± 0.17	13.50 ± 0.30	16.43 ± 0.67	10.03 ± 0.99	11.55a ± 0.60	3.38a ± 0.28	10.03b ± 0.99
	PRD	24.32 ± 0.85	16.86 ± 1.24	18.88 ± 0.40	4.32 ± 0.24	3.90a ± 0.19	2.64 ± 0.09	14.11 ± 0.70	18.44 ± 1.20	14.29 ± 0.52	9.36b ± 0.51	2.58b ± 0.21	14.29a ± 0.52
	DI	23.84 ± 0.28	17.42 ± 1.80	19.50 ± 0.92	3.35 ± 0.06	3.71ab ± 0.11	2.69 ± 0.28	14.64 ± 0.34	18.34 ± 0.54	12.69 ± 0.35	7.60bc ± 0.43	2.49b ± 0.13	12.69ab ± 0.35
SWP	FI	25.32 ± 1.34	13.48 ± 0.38	17.72 ± 0.55	4.45 ± 0.21	3.32bc ± 0.10	2.53 ± 0.06	13.59 ± 0.46	13.97 ± 0.78	11.04 ± 0.31	7.91bc ± 0.40	2.33b ± 0.09	11.04b ± 0.31
	PRD	23.45 ± 0.73	14.33 ± 0.24	20.27 ± 0.74	3.52 ± 0.21	3.21bc ± 0.05	2.81 ± 0.20	14.51 ± 0.32	15.14 ± 0.21	12.15 ± 0.20	6.90cd ± 0.43	2.06bc ± 0.05	12.15ab ± 0.20
	DI	21.41 ± 0.36	15.19 ± 0.55	20.50 ± 0.50	3.75 ± 0.40	3.67ab ± 0.07	2.60 ± 0.27	15.46 ± 0.24	16.92 ± 0.46	11.61 ± 0.67	7.82bc ± 0.46	2.48b ± 0.21	11.61ab ± 0.67
WSP	FI	23.84 ± 0.20	13.17 ± 0.31	14.13 ± 0.83	3.53 ± 0.17	3.25bc ± 0.10	2.14 ± 0.21	14.14 ± 0.65	13.38 ± 0.47	10.04 ± 0.92	6.24cd ± 0.34	1.99bc ± 0.05	10.04b ± 0.92
	PRD	21.41 ± 0.61	13.78 ± 0.25	15.35 ± 0.76	2.98 ± 0.10	2.95cd ± 0.07	2.03 ± 0.09	15.37 ± 0.67	14.84 ± 0.36	9.81 ± 0.61	6.45cd ± 0.22	1.92bc ± 0.16	9.81b ± 0.61
	DI	18.80 ± 0.73	13.22 ± 0.20	17.11 ± 0.69	2.68 ± 0.15	2.65d ± 0.08	2.38 ± 0.08	18.02 ± 0.36	13.90 ± 0.59	11.39 ± 0.99	5.16d ± 0.22	1.56c ± 0.04	11.39ab ± 0.99
Output of two-way ANOVA (*p*-value)													
Biochar (B)		***	***	***	***	***	**	***	***	**	***	***	**
Irrigation (I)		***	ns	**	***	ns	ns	***	**	ns	***	**	**
B * I		ns	ns	ns	ns	**	ns	ns	ns	ns	***	*	*

The treatments are different biochar (Control, SWP, and WSP) and irrigation (FI, DI, and PRD).

Values are the mean ± standard error (n = 4). *, **, and *** indicate significant levels at p< 0.05, p< 0.01, and p< 0.001, respectively. ns indicates no statistical significance. Different letters following the mean indicate significant differences between treatments at the p< 0.05 level by Tukey’s test.

[P]_leaf_, [P]_stem_, and [P]_root_ were affected by biochar addition, where the biochar-added plants had greater [P] in each of the organs than the unamended plants except in root, and plants grown under SWP possessed higher [P]_leaf_, [P]_stem_, and [P]_root_ than those grown under WSP. Irrigation treatment had a significant effect on [P]_leaf_, where reduced irrigation decreased [P]_leaf_, and the reduction was more evident under DI. In addition, there was a significant interactive effect between two main factors on [P]_stem_.

Plants grown under biochar amendment possessed greater [K]_leaf_ than those grown under unamended soils. Plants watered with reduced irrigation had considerably higher [K]_leaf_ than those watered with FI, and had much higher [K]_leaf_ in PRD than in DI. For [K]_stem_, biochar-added plants had superior [K]_stem_ compared to unamended plants, and SWP plants possessed higher [K]_stem_ than WSP. When analyzed across the biochar addition, reduced irrigation especially DI increased [K]_stem_ compared to FI. Moreover, biochar-added plants had higher [K]_root_ compared to unamended plants, especially with WSP amendment.

Plants grown under biochar addition possessed lower [Ca]_leaf_, [Ca]_stem_, and [Ca]_root_ compared to those grown under unamended soils, particularly with SWP amendment. Among the three irrigation treatments, both [Ca]_leaf_ and [Ca]_stem_ were the highest in plants grown under FI, followed by PRD, with DI being the lowest. Conversely, for [Ca]_root_, PRD plants were the highest, followed by DI plants, with FI plants being the lowest.

**Figure 3:**
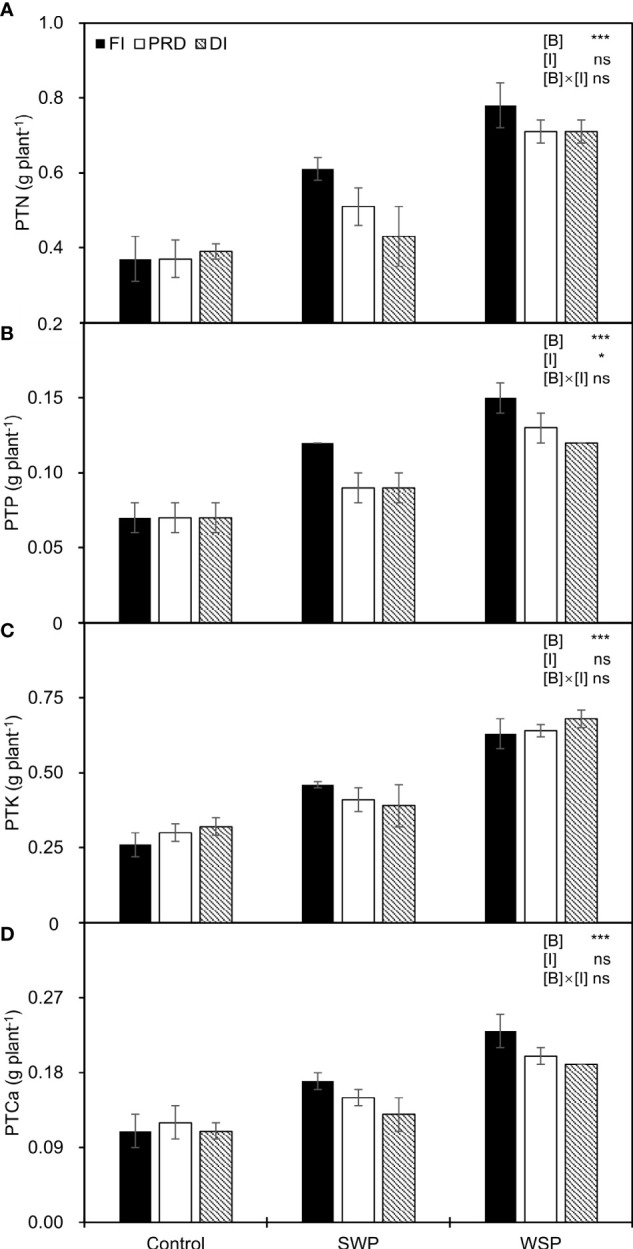
**(A)** Plant total N uptake (PTN), **(B)** plant total P uptake (PTP), **(C)** plant total K uptake (PTK), and **(D)** plant total Ca uptake (PTCa) of maize plants exposed to different biochar (Control, SWP, and WSP) and irrigation (FI, DI, and PRD) treatments. Values are the mean ± standard error (*n* = 4). * and *** indicate significant levels at *p*< 0.05 and *p*< 0.001, respectively. ns indicates no statistical significance.

As expected, PTN, PTP, PTK, and PTCa were significantly affected by biochar treatments (**Figure 3**), being greater on biochar-added plants than on non-biochar plants. Moreover, regardless of biochar amendment, reduced irrigation significantly decreased PTP in relation to FI, and a decreased trend was more pronounced with DI treatment.

### 3.4 Leaf area, plant biomass production, and distribution

Biochar addition especially WSP significantly increased the LA of maize plants than non-biochar controls (**Table 6**). Plants grown under PRD and DI possessed lower LA than those grown under FI, and LA was greater under PRD compared to DI. SLA was significantly affected by biochar treatment (**Table 6**), being lower on biochar-amended plants than on non-biochar plants. LDM was significantly affected by two main factors (**Table 6**). As expected, compared to non-biochar plants, biochar-amended plants had significantly greater LDM. FI plants had higher LDM than reduced irrigation plants, which was slightly greater in PRD than in DI. S_tem_DM, FDM, and S_hoot_DM were affected significantly by biochar addition (**Table 6**), where WSP plants had the highest S_tem_DM, FDM, and S_hoot_DM, followed by SWP plants, with the non-biochar plants being the lowest. Also, the trend of change in RDM was the same for S_hoot_DM (**Table 6**). RSR varied among biochar treatments (**Table 6**), being lower on biochar-amended plants than on non-biochar plants.

**Table 6:** The effects of treatments and output of two-way ANOVA for leaf area (LA), specific leaf area (SLA), leaf dry matter (LDM), stem dry matter (S_tem_DM), shoot dry matter (S_hoot_DM), fruit dry matter (FDM), and root dry matter (RDM) of maize plants.

Biochar (B)	Irrigation (I)	LA (cm^2^ plant^−1^)	SLA (cm² g^−1^)	LDM (g plant^−1^)	S_tem_DM (g plant^−1^)	FDM (g plant^−1^)	S_hoot_DM (g plant^−1^)	RDM (g plant^−1^)
Control	FI	1,124.75c ± 273.28	244.91 ± 6.41	4.59 ± 1.31	10.98 ± 1.86	4.15 ± 1.51	19.72 ± 4.65	2.60 ± 0.54
	PRD	1,294.25bc ± 222.32	170.36 ± 19.03	4.50 ± 0.90	11.43 ± 1.41	5.50 ± 1.42	21.43 ± 3.60	3.56 ± 0.85
	DI	969.50c ± 139.43	232.08 ± 4.46	4.18 ± 0.64	12.17 ± 1.00	5.55 ± 0.87	21.90 ± 2.10	0.85 ± 0.54
SWP	FI	2,032.00bc ± 93.30	219.15 ± 5.44	9.27 ± 0.56	20.15 ± 0.64	9.62 ± 1.85	39.04 ± 2.55	5.83 ± 0.78
	PRD	1,622.50bc ± 151.64	217.42 ± 12.04	7.46 ± 0.81	15.85 ± 1.32	8.70 ± 1.25	32.00 ± 3.33	5.52 ± 0.72
	DI	1,386.00bc ± 230.83	228.62 ± 20.52	6.06 ± 1.28	14.88 ± 2.50	6.72 ± 1.78	27.66 ± 5.50	3.99 ± 0.93
WSP	FI	3,148.25a ± 145.32	197.38 ± 13.42	15.95 ± 0.41	24.21 ± 2.91	23.01 ± 3.75	63.17 ± 1.06	8.07 ± 1.14
	PRD	2,476.00ab ± 109.47	207.25 ± 1.25	14.54 ± 0.97	23.58 ± 0.97	18.61 ± 2.35	56.72 ± 1.55	7.50 ± 0.80
	DI	2,377.65bc ± 136.19	179.89 ± 17.86	13.22 ± 0.63	25.46 ± 1.24	17.78 ± 1.34	56.45 ± 2.73	5.85 ± 0.66
Output of two-way ANOVA (*p*-value)								
Biochar (B)		***	***	***	***	***	***	**
Irrigation (I)		*	ns	*	ns	ns	ns	ns
B * I		*	ns	ns	ns	ns	ns	ns

The treatments are different biochar (Control, SWP, and WSP) and irrigation (FI, DI, and PRD).

Values are the mean ± standard error (n = 4). *, **, and *** indicate significant levels at p< 0.05, p< 0.01, and p< 0.001, respectively. ns indicates no statistical significance. Different letters following the mean indicate significant differences between treatments at the p< 0.05 level by Tukey’s test.

### 3.5 Relationships between different traits of soil–plant systems

The maize leaf N_area_-to-root N_length_ ratio varied among biochar treatments (**Table 7**), being greater on biochar-amended plants than on non-biochar plants, especially with WSP addition. The plants grown in the reduced irrigation treatments possessed higher RLR than those grown in FI (**Table 7**), and RLR was greater under PRD compared to DI. RSR was significantly affected by biochar treatments (**Table 7**), where biochar addition particularly with WSP caused lower RSR compared to the non-biochar controls.

**Table 7:** The effects of treatments and output of two-way ANOVA for [N]_leaf_ per unit leaf (leaf N_area_), [N]_root_ per unit root length (root N_length_), leaf N_area_-to-root N_length_ ratio (leaf N_area_/root N_length_), root area-to-leaf area ratio (RLR), and root biomass-to-shoot biomass ratio (RSR) of maize plants.

Biochar (B)	Irrigation (I)	Leaf N_area_ (g m^−2^)	Root N_length_ (mg m^−1^)	Leaf N_area_/Root N_length_	RLR	RSR
Control	FI	1.03 ± 0.04	1.74 ± 0.68	0.84 ± 0.24	0.54c ± 0.04	0.14 ± 0.04
	PRD	0.91 ± 0.19	0.99 ± 0.10	0.91 ± 0.16	0.77bc ± 0.14	0.16 ± 0.02
	DI	1.03 ± 0.07	1.12 ± 0.16	0.96 ± 0.11	1.09a ± 0.14	0.20 ± 0.03
SWP	FI	1.16 ± 0.07	1.45 ± 0.08	0.81 ± 0.08	0.76bc ± 0.07	0.15 ± 0.01
	PRD	1.07 ± 0.03	1.30 ± 0.13	0.86 ± 0.11	0.82abc ± 0.05	0.17 ± 0.01
	DI	0.91 ± 0.08	1.53 ± 0.32	0.65 ± 0.08	0.65bc ± 0.10	0.14 ± 0.01
WSP	FI	1.11 ± 0.03	1.02 ± 0.10	1.12 ± 0.11	0.62bc ± 0.08	0.13 ± 0.02
	PRD	0.99 ± 0.03	1.14 ± 0.19	1.16 ± 0.09	1.11bc ± 0.23	0.13 ± 0.02
	DI	1.05 ± 0.06	0.86 ± 0.05	1.00 ± 0.18	0.61ab ± 0.09	0.10 ± 0.01
Output of two-way ANOVA (*p*-value)						
Biochar (B)		ns	ns	*	ns	*
Irrigation (I)		ns	ns	ns	*	ns
B * I		ns	ns	ns	*	ns

The treatments are different biochar (Control, SWP, and WSP) and irrigation (FI, DI, and PRD).

Values are the mean ± standard error (n = 4). * indicates significant levels at p< 0.05. ns indicates no statistical significance. Different letters following the mean indicate significant differences between treatments at the p< 0.05 level by Tukey’s test.

**Figure 4:**
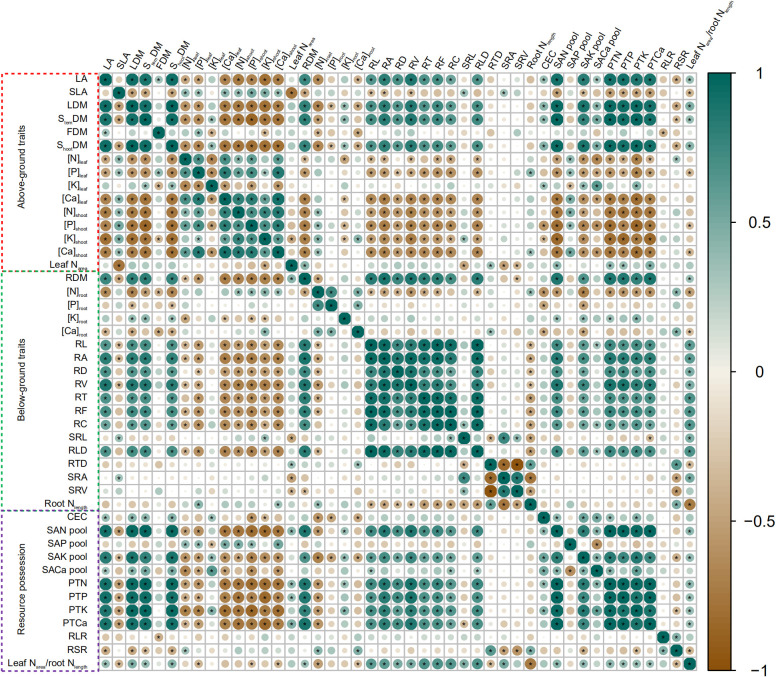
Heatmap of Pearson correlation coefficient between different traits of soil–plant systems across biochar and irrigation treatments. According to the legend, the scale of the circles and the darkness of each box color correspond to *R* ^2^ values; * indicates significant levels at *p*< 0.05. Increase and decrease in abundance are indicated in the colored bar with green and brown, respectively.

All data were divided into aboveground traits, belowground traits, and resource possession for analysis by correlation heatmap. The results are presented in **Figure 4**. RL, RA, RD, RV, RT, RF, RC, and RLD were negatively correlated to N, P, K (except leaf), and Ca concentrations in maize aboveground organs, but were positively correlated to PTN, PTP, PTK, and PTCa. Likewise, SAN pool, SAK pool, and SACa pool were positively correlated to PTN, PTP, PTK, and PTCa. SAP pool negatively affected nutrient concentrations in aboveground organs (except leaf). SRL and SRA were positively correlated to SLA but negatively correlated to leaf N_area_. RDM and leaf N_area_-to-root N_length_ ratio were positively correlated to LA, LDM, S_tem_DM, and S_hoot_DM. RSR was positively correlated to RTD and RLR but negatively correlated to LA, LDM, S_tem_DM, and S_hoot_DM. RLR was negatively correlated to FDM.

**Figure 5:**
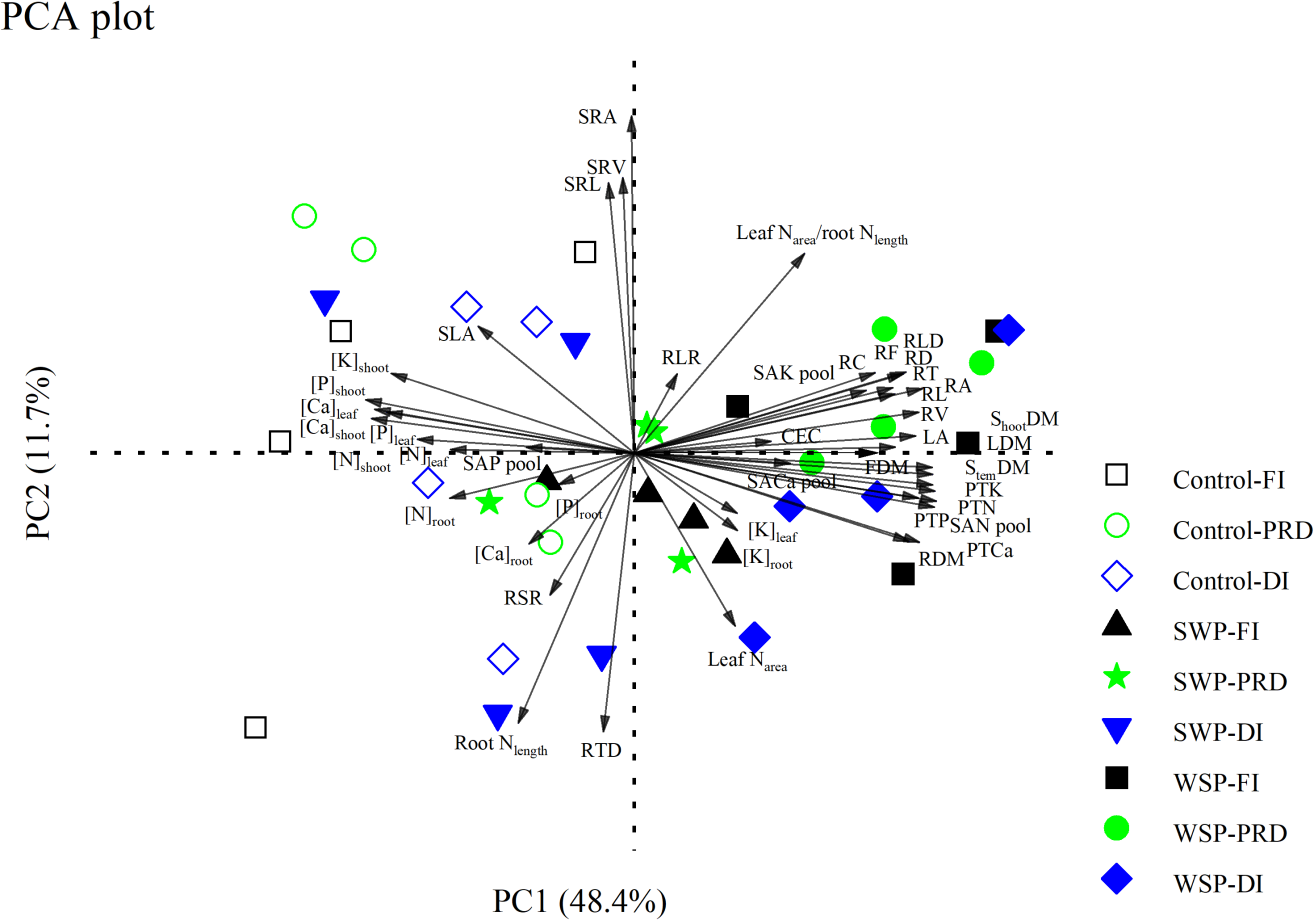
Principal component analysis (PCA) of all the measured parameters on maize plants grown under full (FI), deficit (DI), or partial root-zone drying (PRD) irrigation without (Control) and with softwood (SWP) and wheat-straw (WSP) biochar applied at 2%. The parameters are as follows: aboveground traits (LA, SLA, LDM, S_tem_DM, FDM, S_hoot_DM, [N]_leaf_, [N]_stem_, [P]_leaf_, [P]_stem_, [K]_leaf_, [K]_stem_, [Ca]_leaf_, [Ca]_stem_, and leaf N_area_), belowground traits (RDM, [N]_root_, [P]_root_, [K]_root_, [Ca]_root_, RL, RA, RD, RV, RT, RF, RC, SRL, RLD, RTD, SRA, SRV, and root N_length_), and resource possession (CEC, SAN pool, SAP pool, SAK pool, SACa pool, PTN, PTP, PTK, PTCa, RSR, RLR, and leaf N_area_/root N_length_). The black, blue, and green graphs represent FI, DI, and PRD, respectively. The hollow and solid graphs represent non-biochar controls and biochar additions, respectively.

PCA showed that biochar treatment separated all the measured variables into distinct clusters, while irrigation treatment marginally seemed to have an impact (**Figure 5**). On the PCA plot, PC1 and PC2 explained 48.4% and 11.7% of the variation, respectively. PC1 isolated biochar addition on the right side of the plot; non-biochar control was isolated on the left side of the plot. Specifically, RL, RA, RD, RV, RT, RF, RC, RLD, RLR, CEC, SAN pool, SAK pool, SACa pool, PTN, PTP, PTK, PTCa, LA, leaf N_area_, leaf N_area_-to-root N_length_ ratio, LDM, S_tem_DM, FDM, S_hoot_DM, [K]_leaf_, and [K]_root_ contributed to biochar clustering, while the concentrations of the other nutrients in the aboveground organs, SAP pool, SLA, and RSR strongly facilitated non-biochar clustering.

## 4 Discussion

### 4.1 Biochar and reduced irrigation affected root morphology, nutrient uptake, and their implications in altering shoot growth

The roots are critical in converting and cycling nutrients in the soil–plant system. Numerous studies have demonstrated the effects of biochar on the roots firstly due to its rich microscopic pore structure and physicochemical properties. For instance, biochar addition significantly lowered the soil bulk density while increasing the total porosity, providing ample space for roots to grow and facilitating root penetration and extension (Oguntunde et al., 2008). Biochar tends to be alkaline in nature and may increase the pH of the soil, which is conducive to promoting root development, particularly in acidic soils (Yuan and Xu, 2011). In addition, the salutary effects of biochar on microbiological activities could potentially affect the rhizosphere environment and hence root growth (Warnock et al., 2007). Other researchers have found that biochar may release small molecules such as ethylene or produce hormone-like substances (Fulton et al., 2013), which might affect root secretions, thus stimulating and interfering with root physiological processes. Likewise, in this study, biochar amendment particularly WSP modified root growth with respect to root morphological traits, as indicated by the increased total RL, RA, RD, RV, RT, RF, RC, and RLD, with a consequent increase in root biomass, which was further amplified by PRD regime. As shown previously by Liu et al. (2022), PRD may stimulate root metabolic capacity and root activity by increasing the root N concentration and root C/N ratio; this response may be related to root penetration under alternating wetting/drying cycle of the soil (Chaves et al., 2003). All these effects induced by the PRD regime could contribute to root growth, especially combined with WSP amendment.

Biochar amendment has been observed to have significant effects on the rhizosphere soil nutrient availability. In our present study, compared to the unamended controls, SWP and especially WSP amendment significantly increased SAN pool and CEC. These might be correlated to biochar properties, especially in terms of N element and CEC, where WSP possessed significantly higher values than SWP. The higher CEC in soil under WSP addition could increase the inorganic N content due to the fact that biochar can adsorb more ${\text{NH}}_{4}^{+}$ -N and strengthen the nitrification of ${\text{NH}}_{4}^{+}$ -N into NO_3_-N (Xu et al., 2016). In addition, the increased SAN pool in WSP-amended soil could potentially be interpreted as lower N leaching or higher N immobilization (Oladele et al., 2019), in which soil enzyme plays an important role (Gao and Deluca, 2018). For instance, Song et al. (2020) pointed out that wheat-straw biochar boosted the activities of β-glucosidase and leucine aminopeptidase in maize rhizosphere soil, and Gao and Deluca (2020) found that wood-based biochar improved the relative abundance of bacterial *amoA* gene in the rangeland ecosystem, which contributed to the immobilization of N. Moreover, these superior properties of biochar regarding porosity and specific surface area could enhance nutrient transport through diffusion and/or mass flow (Oladele et al., 2019).

Previous studies have found that soil P availability was connected to pH and that the high pH of biochar is generally caused by metal oxides and carbonates with high P adsorption capacity, such as oxide forms of Ca, Mg, Fe, and Al (Karunanithi et al., 2017); thus, P at neutral pH may be relatively more effective. After mixing biochar and soil, P ion precipitate with free Ca^2+^ and Mg ^2+^ is released from the biochar or co-precipitate with mixed mineral complexes (Al-Si-Fe-Ca) on the biochar surface (Shepherd et al., 2017). In the present study, WSP possesses greater pH and alkali metal ions, and the possible presence of carbonate and calcite might enhance P absorption capacity, hereby reducing SAP pool. Compared to WSP, SWP possessed higher C content, which may result in a potentially higher aromatic C, less crystalline mineral phases, and a more neutral pH. Our previous results reported that P sorption and release may co-occur in SWP addition (Bornø et al., 2018), consistent with the findings obtained here. Therefore, the effect of biochar on SAP depends on the feedstock of biochar.

WSP amendment caused greater SAK pool than the unamended soils, whereas there was no such effect with the addition of SWP, in good agreement with previous results reported by Liu et al. (2022). Generally, biochar possesses ash that can facilitate the electrostatic attraction of K^+^ on the surface of the biochar–soil matrix, thereby reducing K^+^ leaching losses (Liu et al., 2021), and most of the K incidental to biochar can usually be absorbed by plants as available K. It has been shown that biochar could enhance water-soluble and exchangeable K in the soil (Oram et al., 2014). Furthermore, biochar amendment could contribute to the dissolution of soil K-containing minerals through pH-mediated increases in soil microbial biomass and enzyme activity (Rahimzadeh et al., 2015), which consequently increased SAK content. Therefore, in the present study, the greater SAK pool generated with the added WSP than SWP was attributed to the higher ash fraction, pH, and CEC status of WSP, which would stimulate the activities of microorganisms and enzymes engaged in the mobilization and/or metabolic processes of K in soil (Gao and Deluca, 2018).

The SACa pool responded similarly to biochar application as SAK pool, in line with the result reported by Lehmann et al. (2003). The WSP-induced increase in the SACa pool could be attributed to its higher CEC. In other words, the direct cation release from biochar dominated the water-soluble Ca in the mixture, thus affecting Ca availability (Xu et al., 2013). Moreover, the large amount of Ca contained in the WSP with soluble P in the mixture may reduce the leaching and transport of available Ca, thus increasing the bioavailability of Ca in the soil (Major et al., 2009).

In addition to biochar, soil nutrient availability might also be influenced by soil water dynamics. The reduction process in soil moisture could reduce nutrient diffusion from the soil matrix to the soil solution, especially for the less mobile nutrients such as P and K (Ghosh et al., 2020). In the present study, we found that SAP appeared to be less affected by soil moisture, in agreement with the results of our previous study (Liu et al., 2022), which may be related to soil type, irrigation regimes, and drought intensity. Mayakaduwage et al. (2021) reported that this effect was modulated by the form in which P was added, and the addition of inorganic P resulted in a high concentration of labile phosphate after weeks of submergence. Therefore, our findings suggested that the soil available nutrient pools were a better representation of soil nutrient availability than single soil available nutrients, where reduced irrigation treatments significantly lowered soil available nutrient pools besides available Ca. It is known that plants grow more slowly under reduced irrigation than under well-water treatment, and this might minimize plant demand for water and nutrients, leading to a buildup of soil organic C and N with a slowdown decomposition of organic matter (Abadía et al., 2021) and, consequently, a marked decline in microbial activities. These manifestations represent a substantial suppression of soil biological activity under restricted irrigation conditions, which would negatively affect the mineralization process of soil minerals, thereby weakening soil nutrient availability (Wang et al., 2017).

Generally, improvements in soil nutrient availability especially in the case of increased root morphological parameters would facilitate plant accessibility to the available nutrients and consequently increased the accumulation of nutrients in the plant (Gahoonia and Nielsen, 2004). Echoing this, SWP and particularly WSP amendment significantly increased PTN, PTP, PTK, and PTCa uptake, which were positively correlated to root morphology (i.e., RL, RA, RD, RV, RT, RF, RC, and RLD) and soil available nutrient pools (i.e., SAN, SAK, and SACa pool), implying that the modified root morphology and increased soil nutrient availability with biochar amendment could independently and/or synergistically promote plant total nutrient uptake and that the effect of root morphology is relatively more pronounced. These changes in the roots may be a deep-seated mechanism for increased PTP despite the reduction in SAP. Interestingly, previous studies have suggested that nutrient uptake might be positively correlated to plant biomass (Chen et al., 2021). Consistent with this, here, biochar amendment especially WSP increased S_hoot_DM, which was closely associated with plant total nutrient uptake. Overall, plants grown under biochar combined with PRD tended to pursue an acquisition strategy by modifying root traits. Thus, the modified root morphology and increased soil available nutrient pools, and consequently the greater plant nutrients status, might have facilitated the improved shoot growth and yield of maize in biochar-added soils compared to the unamended soils.

Furthermore, soil available nutrients appeared to have strong relationships with root morphological traits. In natural ecosystems, there was evidence confirming their connectedness, with soil nutrient availability being able to adequately nourish the dynamic performance of roots (Vogt et al., 1995). However, our experiment created relatively limited space belowground; thus, whether root traits were dominated by soil available nutrients under such conditions is unclear and needs to be further explored under field conditions.

### 4.2 The allometric relationships between aboveground and belowground traits under biochar and reduced irrigation regimes

Biochar amendment has been shown to modulate leaf development (Liu et al., 2022). Here, we found that the plants grown under biochar addition increased LA but depressed SLA compared to the non-biochar plants; similar findings were shown by Olmo et al. (2014). Furthermore, Niinemets (1999) reported that SLA was negatively correlated to leaf thickness, suggesting a corresponding increase in leaf thickness when SLA was reduced; this enables one to interpret that biochar addition promoted leaf thickness and facilitated plant adaptation to changing water-scarce environments (Galmes et al., 2007).

The allometric relationships of aboveground and belowground traits in response to water and fertility constraints are extremely important for deciphering the strategies of plants to cope with multiple environments (Carmona et al., 2021). For instance, here the SLA was positively correlated to SRL and SRA, which depends on plant competition for specific nutrients and/or light effectiveness (Liu et al., 2017). This may implicate that plant acquisition strategies for resources are synergistic through aboveground and belowground. Moreover, in the present study, the leaf N_area_-to-root N_length_ ratio increased with WSP amendment, which facilitates plants to reduce fine root respiration rate under drought conditions and thereby to increase root longevity (Eissenstat et al., 2000), while maintaining strong internal CO_2_ gradients to offset prolonged stomatal closure (Wright and Westoby, 2002).

The decreased RSR was observed under biochar amendment, especially under WSP. However, this result was not in accordance with our expectation, which may be due to the fact that WSP increased grain yield and resulted in higher total shoot biomass and lower RSR. Therefore, RSR could not objectively represent the actual ratio of water uptake to evapotranspiration in the present study. This may also be an underlying factor in the divergent results of many studies (Turner, 1996; Liu and Stützel, 2004). Therefore, the balance between transpiration and absorption capacity can be more scientifically expressed through the ratio of root absorption area to leaf transpiration area (i.e., RLR). Here, compared to FI, RLR was significantly increased under reduced irrigation treatments, especially under PRD. The response of the RLR to drought might be triggered by the absolute growth of the root structure (Gazal and Kubiske, 2004), which provides a relationship between the surface area of water uptake and transpiration losses (Diaz-Espejo et al., 2012). This suggests a more conservative balance between aboveground and belowground in PRD plants under the same edaphic water limitation.

## 5 Conclusion

Collectively, the results of this study demonstrate that biochar amendment could mitigate the partial adverse effects of reduced irrigation. Particularly, WSP amendment combined with PRD irrigation enhanced soil available N, K, and Ca pool and cation exchange capacity status, with a tendency to engage the plant in an acquisitive strategy by modifying root morphological traits, thereby promoting plant total nutrient accumulation, shoot growth, and yield. In addition, aboveground and belowground traits respond synergistically to abiotic stresses in the environment created by the co-creation of WSP and PRD, which is a considered reliable agricultural strategy from the perspective of maize productivity and soil nutrient availability in the face of both water deficit and soil degradation. However, biochar amendment was found to adversely affect soil available P pool. Accordingly, further research on P cycling in the presence of biochar is needed in the future, especially the long-term or field effects of biochar on soil available nutrients.

## Data availability statement

The raw data supporting the conclusions of this article will be made available by the authors, without undue reservation.

## Author contributions

HW: Experiment design and execution, conceptualization, methodology, investigation, data curation, formal analysis, and writing—original draft preparation. XL: Reviewing and editing. QS: Formal analysis. YC: Data collection. MJ: Software. JZ: Formal analysis. BC: Reviewing and editing. JH: Data analysis. ZW: Experiment execution and funding acquisition. MH: Editing. FL: Supervision, conceptualization, methodology, data curation, funding acquisition, language editing, and validation. All authors contributed to the article and approved the submitted version.
